# Exploring the Effects of Argument Map-Supported Online Group Debate Activities on College Students’ Critical Thinking

**DOI:** 10.3389/fpsyg.2022.856462

**Published:** 2022-05-19

**Authors:** Xinya Chen, Li Wang, Xuesong Zhai, Yan Li

**Affiliations:** ^1^Faculty of Education, Henan University, Kaifeng, China; ^2^College of Education, Zhejiang University, Hangzhou, China

**Keywords:** depth of critical thinking, phases of critical thinking, argument map (AM), number of speeches, college students

## Abstract

Debate has been warranted as a meaningful activity to promote students’ higher-level thinking, such as critical thinking. However, traditional debate activities which are typically carried out in the physical classroom may meet some obstructions of limited time and space, which would result in the phenomenon that many participants act as silent watchers rather than mind exchangers. Moreover, it is hard to make a visualized record about the whole process and contents of the traditional debate activity. The current study aimed to explore the effects of argument map (AM)-supported online group debate activities on college students’ critical thinking, including their depth and phases of critical thinking, and the relationship between students’ depth of critical thinking and their number of speeches. In the study, an innovative argumentation construction way was designed. All students enrolled in a course could have a chance to attend the AM-supported online group debate activities and the whole process and outcomes of online group debate could be visualized by AM. At the same time, the effectiveness of the innovative activities was evaluated by content analysis of AM. A total of 42 sophomores in the undergraduate course named “Learning Sciences and Technology” were recruited to attend online group debate activities assisted by a web-based visualization tool named “ZJU Yuque” in 5 weeks. Newman’s framework about the depth of critical thinking and Murthy’s instrument of critical thinking phases were employed as guidelines. We found that 42 students’ overall depth of critical thinking was gradually improved in an obvious way during the five online debate activities. The most frequent phases for students in the first and second team in online group debate activities include Understand→Understand (U→U), Recognize→Understand (R→U), and Understand→Evaluate (U→E). However, students’ creating behaviors were not significantly generated. Teachers’ real-time feedback was helpful for students’ improvements of high-level thinking skills and their preparation for the next debate activity. Students’ interviews found that students highly valued such online group debate activities because every student could have a chance to express their thoughts and they had enough time to prepare debate contents. Based on the findings, some implications were proposed for the better design and implementation of online group debate activities.

## Introduction

Critical thinking refers to the “purposeful, self-regulatory judgment which results in interpretation, analysis, evaluation, and inference, as well as an explanation of the evidential, conceptual, methodological, criteriological, or contextual considerations upon which that judgment is based” (Facione, 1990). In this media-enriched age, people are confronted with all kinds of information produced by various professional or non-professional producers. Critical thinking skills become more and more important in social contexts where adequate decision-making and problem-solving behaviors are needed (Ku, 2009).

Critical thinking was closely related to the creative thinking (Siburian et al., 2019). Therefore, promoting students’ critical thinking skills is an essential goal of school education (Wang et al., 2007). However, many college students often discussed issues without using sufficient evidence, their critical thinking skills still need to be improved (Rodriguez et al., 2016). Such a phenomenon implicates that traditional lecture was not an effective way to cultivate students’ critical thinking skills (Ahmad et al., 2019).

Such instructional activities as dialogue, free questioning, debate, self-assessed homework, problem-based learning, and collaborative group work were proven efficient in improving learners’ depth of critical thinking (Omelicheva and Avdeyeva, 2008; Carroll et al., 2009; Şendağ and Ferhan, 2009; Fung and Howe, 2014). Among them, the debate is a commonly used method, especially in social science-related courses in universities. The debate is a formal discussion on a particular matter in a public meeting or legislative assembly, in which opposing arguments are put forward, and it usually ends with a vote (Hanna et al., 2014). The debate was proven to positively impact students’ critical thinking, collaboration, engagement, and communication skills (Omelicheva and Avdeyeva, 2008; Hartin et al., 2017; Mitchell, 2019).

Argumentation is the main activity in the process of debate (Clark and Sampson, 2008), it asks debaters make assertions, conclusions, or rebuttals with supportive evidence (Zohar and Nemet, 2002), which could practice their critical thinking skills (Kuhn et al., 2004; Alvarez-Ortiz, 2007). However, in traditional face-to-face debate carried out in the physical classroom, only very few students were selected as debaters to engage in arguments. In contrast, most students only act as audiences without chances to express their thoughts about the topic. This problem was more serious in university courses on a large scale.

Some efforts have been made to ensure that all students can participate in debates. For example, students were asked to write down their arguments, allowing them to present their opinions with more sentences than just the propositions (Monk, 2001; Harrell, 2004). However, the massive amounts of written arguments made it difficult for debaters to assimilate and understand the “gist” of arguments (Kintsch and van Dijk, 1978; Harrell, 2005). Therefore, some measures should be taken to organize those written arguments more logically and clearly. Additionally, it is rare for the instructor to evaluate the debaters’ depth of critical thinking by analyzing their written content on argument, or to explore its relationship with debaters’ participation level, participation level usually measured by debaters’ number of speeches.

To deal with such issues, the aim of this study was to design an argument map (AM)-supported online group debate activity in an undergraduate course, in which all students could attend the debate by constructing an argument map in an online learning platform. Debaters’ critical thinking was objectively evaluated by analyzing the content they wrote in AM. Before introducing the main contents of the study, the literature review was presented.

## Literature Review

### Argument Map and Debaters’ Critical Thinking

An argument map is a visual representation of argumentation; in an AM, claims and evidences were represented by a diagram comprising of “colorful boxes and arrows” and indicates a claim and claim-evidence relationship, colorful boxes indicate the status of basic claims, such as claims, reasons, rebuttals, they are represented by different colors. The arrows reveal the evidence-based relationships between basic claims (van Gelder, 2002; Dwyer, 2011). An AM has some apparent advantages. Firstly, it makes a record about argumentation, and this provides debaters with sufficient time to think and communicate (Klein, 1999). Secondly, it demonstrates argumentation through dual modalities (visual-spatial/diagrammatic and verbal/propositional) to facilitate students’ deeper encoding of argumentation; the deeper encoding of argumentation could practice students’ critical thinking skills (van Gelder, 2003). Thirdly, AM presents information in a hierarchical manner. The hierarchically organized information was proven to promote learning and memory (Dwyer et al., 2012). Practically, although AM was usually constructed in the traditional form by using manuscript and pencil (Alvarez-Ortiz, 2007; Butchart et al., 2009; Dwyer et al., 2011), it has some shortcomings, for example, the inconvenience to edit or change, especially when the map size is large.

To deal with such shortcomings, computer-based AM was proposed as a better argumentation development (van Gelder, 2000). Constructed asynchronously in an online environment, computer-based AM not only provides students sufficient time to explore further information and think deeply (Dracup, 2012), but also allows them to add or delete content freely. Empirical studies have indicated the positive relationship between computer-based AM and students’ critical thinking (Alvarez-Ortiz, 2007; Butchart et al., 2009; Carrington et al., 2011; Harrell, 2011; Eftekhari et al., 2016). For example, Eftekhari et al. (2016) compared the effects of three kinds of instruction (argument mapping instruction *via* Rational software, argument mapping *via* manuscript and pencil, and traditional instruction without AM) on students’ critical thinking. Result demonstrated that students showed the highest level of critical thinking when they constructed AM through Rational software.

Whether constructed in a traditional or computer-based environment, AM focuses on the inferential structure of argumentation and require all argumentation with “boundaries” (Davies, 2011). In another word, AM emphasizes not only evidences, but also the relationship among those evidences. When constructing AM, the Toulmin model is frequently used to ensure the rigorousness and comprehensiveness of argumentation (Toulmin, 1958). The mode has six parts, such as claim, data, warrant, backing, rebuttal, and qualifier. However, the model focuses on “Monolog argumentation” (Schwarz and Baker, 2016), that is, the argumentation is the product of individual thought, ignoring the influence of human interaction. Therefore, the rebuttals, which are considered as the high level of argumentation (Osborne et al., 2004), are often generated by the person who has already made the claim, data, warrant, backing, and qualifier. While in fact, when rebuttals were proposed by those with opposing views, it is supposed to be more efficient in improving students’ depth of critical thinking. However, rarely studies have explored this.

### Debaters’ Number of Speeches and Their Critical Thinking in Argument Map

Generally speaking, when students construct AM, their number of speeches is an important reference to reflect the engagement. The more the number of their speeches, the larger size the argument map. According to the fact that mapping (Eftekhari and Sotoudehnama, 2018) and software (Carrington et al., 2011) could improve learners’ meaningful engagement, computer-based AM, which is usually featured as constructing maps by software, potentials in enhancing students’ active engagement. When students engaged in constructing the correct AM, they were required to select the grounds correctly, make the relationship among premises clearly and conclude completely. All these were beneficial to students’ depth of critical thinking. Dwyer et al. (2010) pointed out that for students skilled in verbal and spatial reasoning; they were likely to engage themselves in reading arguments to practice their critical thinking. Additionally, van Gelder et al. (2004) found that learners’ critical thinking performance and their AM practice hours were significantly correlated in a computer-supported learning environment.

However, some studies found no difference between students’ critical thinking and their engagement (Dwyer et al., 2012), in which the engagement was measured by the number of argument maps students completed. The possible reason may be that students could not reasonably assimilate a large number of argumentation in a short period of time (Dwyer et al., 2010, 2013). In Dwyer’s (2011) study, when the recall was tested, students in smaller argumentation groups outperformed those in larger argumentation groups. It becomes even worse when the topics were not attractive to students. The unattractive topics took students’ more time to assimilate information. Whether the larger size of the maps, the higher depth of students’ critical thinking. Such question still needs to be further explored. And usually, the size of AM could be quantified as the number of debaters’ speeches.

### Evaluation of Debaters’ Critical Thinking in Computer-Based Argument Map

Many previous studies have used the survey to evaluate students’ depth of critical thinking in computer-based AM (Dwyer et al., 2012; Eftekhari et al., 2016; Gargouri and Naatus, 2017). For example, Dwyer et al. (2012) utilized a survey to collect data about the effectiveness of AM on enhancing students’ depth of critical thinking; the AM was constructed in the platform of Rationale. Although results indicated that computer-based AM was efficient in promoting students’ critical thinking by quantitative gains, it is sometimes quite subjective due to the influence of uncertain factors in filling the survey. Open-ended items encouraging students to think multi-directionally and creatively were more adaptable to measure high-level skills (Akay et al., 2006; Bahar et al., 2012). Content analysis was appropriate to analyze the contents students responded in open-ended items.

Content analysis is an objective evaluation method; it usually codes students’ oral or written contents based on the specific theories or frameworks. For example, in the study of Rapanta and Walton (2016), content analysis was used to assess students’ weaknesses in reasoning; 1,230 units of argumentative discourses in AM were coded, the AM was constructed in Rational software, results indicated that weakness usually happened when students constructed rebuttals, or proposed reasons to support a possible counter-argument.

In addition to theories, some frameworks are also frequently used to code students’ oral or written contents. For example, in critical thinking-related studies, Newman’s depth of critical thinking framework (Newman et al., 1995) or Murphy’s phases of critical thinking framework (Murphy, 2004) were frequently utilized. According to Newman’s framework, the depth of critical thinking is indicated by a value, which is between −1 and 1. The larger the value, the higher the depth of critical thinking. According to Murphy’s framework, the phases of critical thinking were divided into five phases, such as recognize, understand, analyze, evaluate, and create. Some previous studies have evaluated learners’ depth of critical thinking by using content analysis based on these frameworks. For example, in order to compare the effects of different interaction strategies on secondary students’ depth of critical thinking, students’ writing reflections were analyzed based on Newman’s framework (Wang and Woo, 2010). Students’ reflections were coded at sentence level. Results demonstrated that when students wrote thoughts individually, they got the highest depth of critical thinking of 0.83. For computer-based AM, however, few studies have applied the frameworks of Newman or Murphy to evaluate debater’s depth and phases of critical thinking.

Literature review has indicated that AM could be adopted to present the structure of argumentation in a clear form. Computer-based AM could provide more participants the opportunities to engage in argumentation activities. However, in previous studies, AM was usually drawn by students in a team holding the same view. Few studies explored the effects of students with opposite viewpoints to draw AM together, which was supposed to facilitate rebuttals. In the study, an innovative argumentation construction way was designed. All students enrolled in a course could have chance to attend the AM-supported online group debate activities and the whole process and outcomes of online group debate could be visualized by AM. As to the effect of AM, many previous studies have proven that AM could promote students’ critical thinking, which was normally evaluated by the survey. It remains unknown that whether or not students could practice their critical thinking by practicing more. In this study, the effectiveness of the innovative debate activities was evaluated by content analysis of AM.

## Purpose of the Study and Research Questions

Based on the literature review, the study aims to design AM-supported online group debate activities to improve college students’ critical thinking skills. In the activities, two debate teams holding the opposite viewpoints could carry out online group debate through drawing AM together. To examine the effects of the activities, not only students’ depth and phases of critical thinking could be evaluated through analyzing the contents in AM, but also the relationship between students’ depth of critical thinking and the number of speeches could be explored. Specifically, the study includes the following research questions:

Q1: In each debate, what are debaters’ general depths of critical thinking and their number of speeches?
Q2: Is debaters’ depth of critical thinking significantly correlated to their number of speeches?
Q3: What are debaters’ phases of critical thinking?
Q4: How do debaters perceive the AM-supported online group debate activities?

## Methodology

### Participants

The participants of this study were 42 sophomores enrolled in Zhejiang University (ZJU), China, which was founded in 1897 and is a top university in China. The 42 participants took the course named *Learning Science and Technology*. This course was a compulsory course for all sophomores majored in education. Among these 42 students, 38 were female and 4 were male. Their average age was 20. To attend the debate activities, these 42 students were randomly divided into five groups. Four groups had eight students and one group had ten students. There were two debate teams (pro and con) in each group, and each team had four or five members. Each debate team was randomly assigned the position (pro or con) of the debate in the group.

### Instructional Design

The course was taught in 8 weeks of Spring Semester 2021 (from 2 March to 22 April 2021). The course was twice a week (one on Tuesday and the other on Thursday). In each lesson, students needed to take two continuous classes and each class lasted 45 min. The main contents of the course include foundation of learning science, technology-supported learning, technology-supported teaching, methodology in learning science studies, learning assessment, and future learning. Students’ performance in the course was mainly assessed from these aspects: posting debate topics (10), debate activities (50), group presentation about debate activities (20), and learning reflection (20).

In the first week, the instructor gave students a general introduction about course arrangement, such as course goals, outline of course contents, course schedule, guidelines for debate, and criteria for students’ performance assessment. The first 2 weeks were featured by the instructor’s lecture about the basic knowledge about learning science. From the 3rd week to the 7th week, five debates were integrated into the course, and it took place on every Thursday. In the last week, students were asked to make a learning reflection.

In the first week, eight rules about the debate were presented to students: (1) all statements and responses should be expressed appropriately and respectful; (2) information should be clear, accurate, and comprehensive; (3) rebuttal should be clear, relevant, and strong; (4) facts, statistics, and literature are needed when supporting opinions; (5) contents should be organized logically, and have reasonable and orderly arrangement; (6) debaters should understand the topic clearly, and debate under the same concept; and (7) information should be presented professionally. By learning these rules, students would better understand the key points of debate.

From the 3rd to the 7th week, on each Tuesday, the instructor lectured on a specific topic. Right after the instructor’s lecture, students were encouraged to propose debate topics through an online discussion board in an online platform named “Learning in ZJU.” After the debate topics were proposed, students were selected their topics through voting. The top three topics were considered as debate topics, and students chose one from the three topics. Table 1 presented the selected debate topics in the course.

**TABLE 1:** Debate topics in the five weeks (from the 3rd to the 7th week).

Week	Course content	Debate topics	
		Pros	Cons
3	Technology-supported learning	Technology narrowed educational gap among regions	Technology widened educational gap among regions
4	Technology-supported teaching	The advantages of education industrialization outweigh its disadvantages	The disadvantages of education industrialization outweigh its advantages
		Pedagogy should be an undergraduate major	Pedagogy should not be an undergraduate major
5	Methodology in learning science studies	More should be done to develop students’ skills in non-continuous texts	More should be done to develop students’ skills in long and continuous texts
		Preschool education should be included in compulsory education	Preschool education should not be included in compulsory education
6	Learning assessment	More general teachers are needed in primary schools	More specialist teachers are needed in primary schools
		In primary education, art education should be based on formal curriculum	In primary education, art education should be based on informal curriculum
7	Future learning	In junior high school, students of different learning levels should be taught in separate classes	In junior high school, students of different learning levels should not be taught in separate classes

**FIGURE 1:**
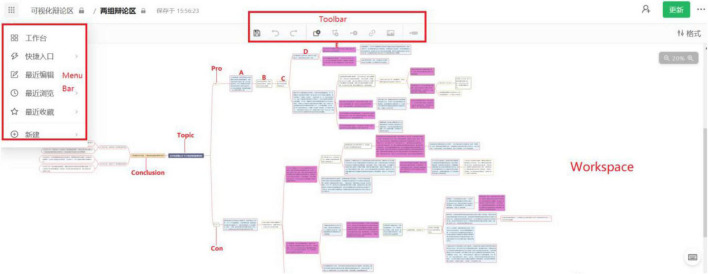
Example of an AM-supported online group debate activity. Reproduced with permission from Zhejiang University.

After selecting debate topics, each group consisting of one pro team (4 or 5 members) and one con team (4 or 5 members) would carry out AM-supported online debate activity through “ZJU YuQue” platform. Comparing with traditional debate activity, the activity designed in this study has obvious innovation in two dimensions. Firstly, all students could have a chance to attend the online debate activities at their own pace through “ZJU YuQue” platform. “ZJU YuQue” is an online collaboration platform that could support students to draw AM at any time. As Figure 1 shows, the platform includes three main sections: menu bar, toolbar, and workspace. The menu bar includes such functions as providing guidance for drawing AM, creating a new AM, viewing recently edited contents, returning back to the previous page, and saving favorite contents. The toolbar could help students add rectangles with different colors. Workspace is the place where students draw their AM. Secondly, the argumentation would be carried out in a visualized way through AM, which allows both sides of a debate to put their viewpoints together and finally draw an AM together. In an AM, a rectangle could contain a speech. In order to standardize the argumentation, rules about colors used in AM were set before the activities were carried out. Burgundy represents rebuttals supported by evidence. Blue represents supplements supported by evidence. White represents rebuttals or supplements without evidence. Students could write their thoughts about argumentation in rectangles. Therefore, students’ viewpoints (the supplement and the rebuttal) and whether they are evidence-based could be easily recognized by different colors. Additionally, the recorded contents could be analyzed to evaluate students’ critical thinking objectively.

Figure 1 presented an example. The selected topic was “technology could narrow/widen educational gaps among regions.” The pro side thought that technology could narrow the educational gap among regions, while the con side thought that technology would widen the educational gaps among regions. A part of the argumentation was listed as following. A, B, C, D, and E in Figure 1 presented some students’ viewpoints. A, C, and D were from the pro side and B and E were from the con side.

(A) Yunnan Luquan No. 1 Middle School, one of the 248 middle schools in poverty-stricken areas, took the synchronous classes through live video with Chengdu No. 7 Middle School, which is a school with high quality of education. This had increased enrollment in poor areas and reduced the loss of local students. In 2019, all students in one class that adopted this teaching method went to key universities.
(B) This is peer-to-peer supporting. The limited high quality resources could not meet the needs of all schools in poor areas.
(C) This problem can be addressed through the flexible application of technology.
(D) The high quality resources could be one-to-many in asynchronous class, such as massive open online courses (MOOC).
(E) Some studies have explored the effect of MOOC on students’ learning. Results indicated that children from poor families showed lower participation and completion rates in MOOC.

The argumentation above demonstrated that the pro side supported their viewpoint with evidence of A. Then A was rebutted by B, which was generated by the con side without evidence. C means the rebuttals to B without evidence. D was rebutted by the con side with evidence, and the evidence was shown as E.

Between the 3rd and the 7th week, on each Thursday, all the five groups were asked to present the main content of their AM. The final score of each debate was decided by the instructor and the teaching assistant.

In the 8th week, some students were randomly invited to share their learning reflections. After the 8th week, all students were required to submit their learning reflections to the instructor. Additionally, students’ semi-structure interview was also carried out 1 week after the 8th week.

### Instrument

In this study, the content analysis framework of critical thinking developed by Newman et al. (1995) was adopted to evaluate debaters’ depth of critical thinking. The framework mainly contained ten indicators and its detailed information is shown in Table 2. According to the framework, an indicator would be marked with “ + ” for positive information if it meets the standard. Otherwise, it would be marked as negative information “−.” Finally, an individual’s depth of critical thinking would be presented by the critical thinking ratio, which is calculated by (X^+^ − X^−^)/(X^+^ + X^−^), and its range is between −1 and 1. The higher the value of the ratio, the higher students’ depth of critical thinking. For example, −1 means that all indicators of an individual’s viewpoints are negative information, and it implicates that the individual has no depth of critical thinking. 1 means that all indicators of an individual’s viewpoints are positive information, it implicates that the individual has a high depth of critical thinking.

**TABLE 2:** Newman’s critical thinking framework (Newman et al., 1995).

Category	Indicator and description	Category	Indicator and description		
Relevance (R ±)	R+	Relevant statements	Ambiguities (A ±)	AC−	Confused statements
	R−	Irrelevant statements, diversions		A+	Clear up ambiguities
Importance (I ±)	I+	Important points/issues		A−	Continue to ignore ambiguities
	I−	Unimportant, trivial points/issues	Linking ideas, Interpretation (L ±)	L+	Linking facts, ideas, and notions
Novelty (N ±)	NP+	New problem-related information		L+	Generating new data from information collected
	NP−	Repeating what has been said		L−	Repeating information without making inferences or offering an interpretation
	NI+	New ideas for discussion		L−	Stating that one shares the ideas or opinions stated without further or adding any personal comments
	NI−	False or trivial guidance	Justification (J ±)	JP+	Providing proof or examples
	NS+	New solutions to problems		JS+	Justifying solutions or judgments
	NS−	Accepting the first offered solution		JS+	Discussing advantages and disadvantages of solution
	NQ+	Welcoming new ideas		JP−	Irrelevant or obscuring questions or examples
	NQ−	Squashing, putting down new ideas		JS−	Offering judgments or solutions without explanations or justification
	NL+	Learner brings new things in		JS−	Offering several solutions without suggesting which is the most appropriate
	NL−	Dragged in by tutor	Critical assessment (C ±)	C+	Critical assessment or evaluation of own or others’ contributions
Bringing outside knowledge or experience to bear on problem (O ±)	OE+	Drawing on personal		C−	Uncritical acceptance or unreasoned rejection
	OC+	Refer to course material		CT+	Tutor prompts for critical evaluation
	OM+	Use relevant outside material		CT−	Tutor uncritically accepts
	OK+	Using previous knowledge	Practical utility (P ±)	P+	Relate possible solutions to familiar situations Discuss practical utility of new ideas
	OP+	Brought course-related problems			
	OQ+	Welcoming outside knowledge		P−	Discuss in a vacuum (treat as if on Mars) Suggest impractical solutions
	OQ−	Squashing attempts to bring experience in outside knowledge			
	O−	Sticking to prejudice or assumptions	Width of understanding (W ±)	W+	Extensive discussion (discuss as a whole)
Ambiguities (A ±)	AC+	Clear, unambiguous statements		W−	Narrow discussion (fragments or parts)

Additionally, the content analysis framework of critical thinking developed by Murphy (2004) was adopted to evaluate the debaters’ phases of critical thinking. According to the framework, the phases of critical thinking could be divided into five phases, namely, recognize, understand, analyze, evaluate, and create (Table 3).

**TABLE 3:** Murphy’s phrases of critical thinking framework (Murphy, 2004).

Process	Descriptor	Code
Recognize	Recognizing or identifying an existent issue, dilemma, problem, etc	R
Understand	Exploring related evidence, knowledge, research, information, and perspectives	U
Analyze	Seeking in depth clarification, organizing known information, identifying unknown information, and dissecting the issue, dilemma, or problem into its fundamental components	A
Evaluate	Critiquing and judging information, knowledge, or perspectives	E
Create	Producing new knowledge, perspectives, or strategies and implementing them or acting on them	C

### Data Collection and Analysis

Data were collected in an innovative way. Different from the survey, which was subjective, the content of AM drawn in “ZJU YuQue” platform was the most important source of data in the study. The contents were analyzed based on the analysis unit. Each rectangle was considered as an analysis unit. Guided by Newman’s depth of critical thinking framework, all analysis units were independently coded by two doctoral students majoring in educational technology.

In order to know the phases of debaters’ critical thinking, the content of AMs in the first- and second-team (the depth of critical thinking ranks in the middle among the ten debate teams) was analyzed. The content was analyzed based on the analysis unit. Each sentence was considered as an analysis unit. Guided by Murphy’s critical thinking phases framework, all analysis units were independently coded by two doctoral students majoring in educational technology. And then the codes were analyzed by the software of GSEQ (short for Generalized Sequential Querier), which is a computer program for analyzing sequential observational data.

In order to understand how students perceive AM-supported online group debate activities, the semi-structured interview was conducted and it was designed by instructor and assistant. The semi-structured interview includes three questions: (1) How do you perceive the online environment? (2) How do you perceive AM? and (3) How do you evaluate instructor’s role during the debate?

## Results

### Debaters’ General Depth of Critical Thinking and Their Number of Speeches in Each Debate

As to Q1 (In each debate, what are the debaters’ general depths of critical thinking and their number of speeches?), the reliability of coding was 0.77, indicating that the coding was reliable. Content analysis found that, in five debates, debaters’ depths of critical thinking were 0.81, 0.85, 0.86, 0.89, and 0.90, respectively, which indicates a gradual upward trend (Table 4). Additionally, debaters’ numbers of speeches from 1th to 5th debate were 212, 329, 293, 361, and 321, respectively. Totally, 1,516 speeches were generated in five debate activities.

**TABLE 4:** Debaters’ depth of critical thinking and their number of speeches in five debates.

Debate	Debaters’ general depth of critical thinking	Debaters’ number of speeches
1	0.81	212
2	0.85	329
3	0.86	293
4	0.89	361
5	0.90	321

### Relationship Between Debaters’ Depth of Critical Thinking and Their Number of Speeches

As to Q2 (Is debaters’ depth of critical thinking significantly correlated to their number of speeches?), correlation analysis found that there was no significant correlation between debaters’ depth of critical thinking and their number of speeches (Table 5). That is, debaters’ depth of critical thinking was not significantly related to their number of speeches.

**TABLE 5:** Correlation analysis of debaters’ depth of critical thinking and number of speeches.

		Debaters’ number of speeches	Debaters’ depth of critical thinking
Debaters’ depth of critical thinking	Pearson correlation	1	0.839
	P		0.076
	N	1516	1516
Debaters’ number of speeches	Pearson correlation	0.839	1
	P	0.076	
	N	1516	1516

### Debaters’ Phases of Critical Thinking

As to Q3 (What is debaters’ phases of critical thinking?), the content of AM was drawn by debaters from the first- and second team were analyzed as a sample. The reliability of the coding was 0.72, indicating that the coding was reliable. Table 6 presents the number of effective single sequences and the top three effective single sequences in each of the five debate activities.

**TABLE 6:** Debaters’ effective single sequences in the first and second team in five debates.

Debate topics	Number of effective single sequences	The top three effective single sequences (number)
1. Does technology narrow educational gap among regions?	92	R→U(18), U→U(18), R→R(13)
2. Does education industrialization outweigh its disadvantages?	141	R→U(23), U→U(22), U→A(14)
3. Which kinds of skills should students develop, non-continuous texts or long and continuous texts?	82	U→U(26), R→U(14), U→ E(8)
4. Which is more needs in primary schools, general teachers or specialist teachers?	100	U→U(31), R→U(14), U→ E(11)
5. In junior high school, should students of different learning levels be taught in separate classes?	137	U→U(31), R→U(24), U→ E(23)

*R, recognize; U, understand; A, analyze; E, evaluate; C, create.*

As for the number of effective single sequences, it was larger in the second- and fifth debates, with the number of 141 and 137, respectively. In these two debates, the type of debate topics was “yes or no,” while in the third and fourth debates, the type of debate topics was “choose one from two,” the former brings a wider range of discussions, while the later limited discussion scopes, which may make the argumentation challenging to carry out. While for the first debate, although the topic type was “yes or no,” students were not familiar with the AM-supported online group debate activities; therefore, the number of effective single sequences was still small.

In terms of the top three effective single sequences, it could be seen that Recognize→Understand (R→U) and Understand→Understand (U→U) ranked first and second in the first and second debate, while they ranked second and first in the last three debates, indicating that the quality of debaters’ critical thinking phases was improved.

Table 7 shows all the sequences generated by debaters in the first- and second team in five debates totally. As Table 7 indicates, in the five debates, debaters in the first- and second team totally generated 552 effective single sequences. The top three effective single sequences such as Understand→Understand (U→U), Recognize→Understand (R→U), and Understand→Evaluate (U→E), with the number of 128, 93, and 50, respectively. There is no sequence to create.

**TABLE 7:** Frequency of effective single sequences in the first and second team in five debates.

	R	U	A	E	C	Total
R	36	93	24	5	0	158
U	45	128	42	50	0	265
A	16	28	11	13	0	68
E	12	31	10	8	0	61
C	0	0	0	0	0	0
Total	109	280	87	76	0	552

*R, recognize; U, understand; A, analyze; E, evaluate; C, create.*

Table 8 presents the adjusted residuals calculated by GSEQ. There were no significant sequences with adjusted residuals greater than 1.96. That is, among the 552 single sequences, there was no significant single sequence, indicating that although some sequences are more or fewer than other sequences, it was not statistically significant.

**TABLE 8:** Adjust residuals of effective single sequences in the first and second team in five debates.

	R	U	A	E	C
R	0.26	0.02	0.82	<0.01	−1.00
U	0.12	0.27	0.96	<0.01	−1.00
A	0.40	0.09	0.92	0.17	−1.00
E	0.99	0.99	0.89	0.88	−1.00
C	−1.00	−1.00	−1.00	–1.00	−1.00

*R, recognize; U, understand; A, analyze; E, evaluate; C, create.*

### Debaters’ Feeling About AM-Supported Online Group Debate Activities

As to Q4 (How do debaters perceive the AM-supported online group debate activities?), semi-structured interview was utilized to collect opinions from 20 interviewees. Table 9 presents the themes, codes, and code-related frequencies generated from the analysis of interview data.

**TABLE 9:** Codes and frequencies of the semi-structured interview data.

Theme	Codes	Frequencies
Feeling of online environment	More time to think	16
	Engage at any time	13
	Rebut without awkwardness	10
	Take up a lot of time	10
	Unable to support or rebut timely	9
Feeling of AM	Get a clear picture of debate	20
	Topic-related support or rebuttals	16
	Evidence-based support or rebuttals	15
	Logical thoughts	13
Feeling of instructor’s role	Evidence-based argumentation	16
	Recognized effort	12
	Gain argumentation skills	9
	Gain professional knowledge	9
	Rebut under the same concept	6

For the question of “How do you perceive the online environment,” interviewees showed two opposite attitudes. Some students expressed their positive attitude. They reported that online environment could provide them more time to think, allow them to engage at any time and rebut without awkwardness. This indicated that AM-supported online debate not only provided all students the flexible time to engage, but also helped them to debate freely without pressure from social relationships through rebutting anonymously. As an interviewee stated, “In online environment, I needn’t end in a specified time. I can add new content in a few days, which deepens my discussion and helps me better understand the topics.” Such statement reflected that the time was sufficient for students to debate online. However, some students expressed the negative attitude about the online environment. They thought that the online environment extended argumentation time and let them spend too much time on the activity. Sometimes, they were unable to support or rebut timely. This implied that the appropriate debating time as well as the reminder whenever students write their speeches in the AM should be set in advance. For example, an interviewee mentioned, “I feel like that I have to argue every day, which took up a lot of time away from doing other things.” Such expression suggested that the online debate was a time-consuming process.

For the question of “How do you perceive the AM,” all of the 20 students expressed their positive attitudes. The reasons might be as follows. The AM helped them to get a clear picture of debate, provide topic-related supplements or rebuttals, make the evidence-based supplements or rebuttals, and organize the thoughts in a logical way. This indicates that the application of AM could not only facilitate students’ evidence-based supplements and rebuttals, but also encourage them to organize these evidences logically. An interviewee stated, “The map makes the argumentation structure and content clear. The different colors represent different means, I could see what others rebut or support my side clearly,” this statement was the indication that students understand the structure of debate in a clear way.

For the question of “How do you evaluate the instructor’s role during the debate,” all the 20 students expressed their positive attitude. Students’ AM was commented and their effort was recognized by the instructor. The instructor emphasized that argumentation should be evidence-based and both teams’ argumentation should be under the same concept. Generally speaking, the instructor’s guidance helped students in gaining both argumentation skills and professional knowledge. For example, an interviewee mentioned, “Evidence-based argumentation was one of the most important words I learned from the instructor’s evaluation. That is, when making a point, we should be sure to back it up.” The sentence showed that students realized the importance of evidence in argumentation.

## Discussion

The aim of the study was to explore the effects of AM-supported online group debate activities on college students’ depth of critical thinking. Five AM-supported online group debate activities were designed based on the platform of “ZJU YuQue.” A total of 42 sophomores majoring in education were invited to attend the debate activities. Guided by Newman and Murphy’s framework of critical thinking, content analysis of debaters’ speeches found some interesting findings.

Firstly, in five debates, debaters’ general depth of critical thinking increased gradually. The result was in agreement with the previous studies (Carrington et al., 2011; Harrell, 2011; Eftekhari et al., 2016), which indicated a positive relationship between computer-based AM and students’ critical thinking. The possible explanation for such a positive relationship may be closely related to the innovative design in this study. (1) Online environment has such advantages as providing all students the chances to engage and letting them have sufficient time to think, write, and edit. It could also reduce the social anxiety through the anonymity function (Maurino, 2006). A similar opinion was expressed by interviewees in the interview. (2) Mapping out argumentation in a visualized way, such as AM, allows students to extract propositions, claims, and rebuttals easily (Chandler and Sweller, 1991; Pollock et al., 2002). The AM helps debaters to identify essential issues, realize their possible lacking of sufficient evidence in supporting their views, make better connections between claims and evidence, all of which were beneficial to facilitate the students’ reflection about reasoning and clarify their insights (Van Gelder, 2005; Kaeppel, 2021), and finally practice their high-level thinking skills (Kabataş, 2011). Such opinions were also verified by students’ interviews. (3) The instructor’s objective and timely feedback on the students’ presentation could help students to reflect and think deeply. In the interview, students expressed that evidence-based, a word usually emphasized by the instructor, was what impressed them most.

Secondly, debaters’ depth of critical thinking was not significantly correlated to their number of speeches. The result was consistent with Dwyer et al. (2012), in which the result indicated that no matter debaters engaged more or less in debate, no difference was found on their critical thinking ability. However, the result was different from the opinion that students’ active participation in argumentation could promote their conceptual understanding (Nam et al., 2011; Kabataş and Çakan, 2020). The conceptual knowledge may facilitate the meaningful learning, which is considered as high quality and efficient learning with active thinking. The difference might be explained from the following perspectives. (1) In this study, students were informed that an important criterion for evaluating AM was its size and shape, the larger size was better. Although the quality of content was also important for the evaluation, they couldn’t be seen intuitively. Students may ignore the quality of argumentation, focusing on increasing their number of speeches to enlarge the map size. From this perspective, evaluation criteria for online debate are suggested to clearly communicate to debaters before debate activity. That is, both the number and the quality of speeches were considered in evaluating online debates. (2) Debate topics were proven to affect students’ critical thinking; the interesting topics motivate students to participate in argumentation (Garcia et al., 1992). In this study, topics were proposed and selected by students themselves. Although some topics were interesting, they were difficult to debate. This was verified by some students in interviews. For example, for the topic of “Should Education be an undergraduate major?,” students expressed that they could not find materials to support their argumentation. They usually used subjective feelings or experiences as evidences. Some debate topics may affect students’ motivation negatively, and therefore, affect their depth of critical thinking. In this view, the instructor may play a more important role in guiding students’ efforts into activities that require more critical thinking (Kaeppel, 2021). (3) Debate aims to refute others with evidence. Therefore, debaters’ opinions are often criticized openly, which might make them feel threatened or defensive (Walker, 2017). The “negative emotional response” may affect critical thinking in a negative way. Therefore, instructors should try to elicit students’ positive emotions when they are involved in the debate. Considering that everyone owns privacy, one efficient way was to be anonymous in online debate; no one knows who is being criticized but himself/herself.

Thirdly, through exploring debaters’ phases of critical thinking in the first- and second team in five debates, it was found that Understand→Understand (U→U), Recognize→Understand (R→U), and Understand→Evaluate (U→E) were the top three effective single sequences, indicating that the phases of debaters’ critical thinking were relatively low. This result could be explained from the following aspects: (1) Butchart et al. (2009) has proven that when students constructed their AMs online, the automatic and real-time feedback was efficient in improving their critical thinking because it provided students opportunities to evaluate and reflect on their own thinking (Dwyer et al., 2012). While in this study, although the instructor provided timely feedback to students’ presentations of debate, due to the limited time in class, it was not fully benefit to students. Therefore, showing students the process of how teachers evaluated their depth of critical thinking based on their speeches, making it as material to study in the next class would be a useful way for students to evaluate their debate. Understanding and assessing argumentation in AM were proved to be effective in improving students’ critical thinking (Carrington et al., 2011). (2) the topics were not closely related to content lectured in class. Therefore, students may be not familiar with the argumentation content, which increased the difficulty of their in-depth analysis of the topics. This was verified by some students’ interview. (3) Although constructing AM in a collaborative manner facilitated students to work together, the uneven participation in groups (Kaeppel, 2021), which manifested as some students kept silent while some were responsible for map construction, may prevent students from working collaboratively or thinking deeply. In this study, this problem was also mentioned by students in interviews. From this perspective, using intelligent technology to automatically record students’ procedural performance, such as their number of speeches, may be effective in promoting students to participate in debate deeply.

## Conclusion, Limitations, and Suggestions

Traditional AM is usually drawn by students on the same side at the fixed time, which is not good for triggering the rebuttals. At the same time, the effects of AM-supported argumentation activities are normally measured by the survey instrument. This study designed an AM-supported online group debate activity, which could allow all of the participants (both the con and the pro sides) to visually debate by drawing online AM together at a flexible pace. Students could not only support their own viewpoints, but also rebut the opposite sides in the same online AM. Students’ depth and phases of critical thinking could be objectively evaluated by analyzing the contents they created in the AM.

Additionally, in order to know whether students’ critical thinking skills were practiced through participating debate, the relationship between students’ depth of critical thinking and the number of speeches was explored in the study. Results indicated that debaters’ general depth of critical thinking was increased gradually. Understand→Understand, Recognize→Understand, and Understand→Evaluate were the top three behaviors for students in the first- and second team usually had during the argumentation. Students’ critical thinking skills were not practiced by participating in the debate.

Based on the results, some enlightenments are proposed. Firstly, the debatable and lecture-related debate topics are the premises for high-quality argumentation. Secondly, drawing AM online allows all students to think and collect debate-related evidence in sufficient time, to express their ideas at their own pace, to organize the evidence in a more logical way, and to understand the structure of argumentation in a more clearly way. Thirdly, rebutting anonymously let participants express themselves freely, for they don’t have to consider the pressure from the social relationship. Fourthly, the timely and procedural feedback from the instructor facilitates students’ gaining in professional knowledge as well as argumentation skills. Finally, in order to practice students’ critical thinking skills through their participation in debate, an evaluation criterion that emphasizes both on the quantity and the quality of debaters’ speeches is needed.

Although the study found some meaningful findings, the study has some limitations. Firstly, the study was carried out in a relatively short period. Secondly, the debate teams included different number of members (4 or 5), this might affect the debate team’s overall depth of critical thinking. Thirdly, some topics were not suitable for debate; the unsuitable debate topics may also affect the debaters’ depth and phases of critical thinking.

Based on the findings and the limitations of the study, some suggestions are put forward. Firstly, in AM-supported online group debate activities, to further explore debaters’ depth and phases of critical thinking, and the relationship between debaters’ depth of critical thinking and their number of speeches, more studies are encouraged in more courses from diverse disciplines. Secondly, the instructor may engage in the process of students proposing and choosing debate topics, ensuring that the topics were closely related to course content taught in class. Finally, considering the familiarity among team members may weaken their frequency of rebuttals, further studies may explore debaters’ depth and phases of critical thinking in an anonymous way.

## Data Availability Statement

The original contributions presented in the study are included in the article/supplementary material, further inquiries can be directed to the corresponding author.

## Author Contributions

XC is mainly responsible for the implementation of experiment, manuscript writing, and data coding. LW is mainly responsible for data coding. XZ is mainly responsible for manuscript writing. YL is mainly responsible for the implementation of experiment and manuscript writing. All authors contributed to the article and approved the submitted version.

## Conflict of Interest

The authors declare that the research was conducted in the absence of any commercial or financial relationships that could be construed as a potential conflict of interest.

## Publisher’s Note

All claims expressed in this article are solely those of the authors and do not necessarily represent those of their affiliated organizations, or those of the publisher, the editors and the reviewers. Any product that may be evaluated in this article, or claim that may be made by its manufacturer, is not guaranteed or endorsed by the publisher.
